# Simultaneous Determination of Metformin, Nateglinide and Gliclazide in Pharmaceutical Preparations Using Micellar Liquid Chromatography

**Published:** 2012-06

**Authors:** Dalia Rashad El-Wasseef

**Affiliations:** Department of Medicinal Chemistry. Faculty of Pharmacy, Mansoura University, 35516, Mansoura, Egypt

**Keywords:** antidiabetic drugs, micellar chromatography, pharmaceutical preparations

## Abstract

A micellar chromatographic method has been developed and validated for simultaneous separation and determination of metformin(MF), nateglinide (NT) and gliclazide (GL). The separation was carried out using a Nucleosil C18 column, 150 mm × 4.6 mm i.d., 5 μm particle size, using micellar mobile phase consisting of sodium dodecyl sulphate (SDS), n-propanol with UV detection. The optimum conditions for the simultaneous separation of the three drugs were 0.12 M SDS, 10% (v/v) n-popanol, 0.3% triethylamine adjusted to pH5.6 with a flow rate of 1 ml.min^-1^ and detection at 254 nm. The limit of detection (LOD) of MF, NT, GL were 0.047, 0.00115, 0.036 μg.mL^-1^ respectively. The method showed good linearity in the ranges of 0.4-16 μg.mL^-1^, (r^2^=0.999), 0.8-16 μg.ml^-1^ (r^2^=0.999) and 1-40 μg.ml^-1^ (r^2^=0.999) for MF, NT, GL respectively. The suggested method was successfully applied for the analysis of the three antidiabetic drugs in pharmaceutical preparations with average recoveries of 99.66%, 100.08% and 100.31% for MT, NT and GL respectively. The results obtained were in good agreement with those obtained from comparison methods. The method was validated regarding accuracy and precision.

## INTRODUCTION

Type 2 diabetes is a long term metabolic disorder where the body becomes resistant to the effect of insulin, a hormone that regulates sugar absorption (1). Treatment of type 2 diabetes (non-insulin dependent) is now possible with orally administered hypoglyceamic agents that help to reduce blood sugar levels. Five main classes of chemically diverse hypoglycemic drugs with different mechanisms of action have been developed for administration to patients. These are known as biguanides (metformin), sulfonylureas (gliclazide, glipizide, glimepiride) thiazolidinedione (pioglitazone, rosiglitazone), meglitinides (natiglinide and repaglinide) and alpha glycosidase inhibitors (acarbose and miglitol) (2). Oral hypoglycemic drugs prescribed as monotherapy have not provided enough hypoglycemic control of type 2 diabetes patients. In such cases a combination of metformin and one of the sulfonyl ureas or nateglinide is used for achieving satisfactory blood glucose levels (3-5).

Available reports suggest that Liquid chromatography-mass spectrometry (LC-MS) and LC with UV detection in conjunction with solid-phase extraction (SPE) or liquid-liquid extraction (LLE) are most commonly used methods for detection and quantitation of antidiabetic drugs (6-13). Based upon the need of co-administration of hypoglycemic agents, several multiscreening procedures have been developed. The reported methods are applicable to analysis of human plasma, urine and tablet formulations (14-18). Micellar liquid chromatography (MLC) (19), which use a surfactant solution with a concentration above critical micellar concentration as a mobile phase, constitutes an alternative to conventional HPLC (20, 21). The simultaneous elution of hydrophobic and hydrophilic analytes is possible (22) without the need for a gradient elution, and direct injection of physiological samples become feasible due to the solubility of proteins in the micelles. The compatibility with conventional reversed-phase column packing is particularly attractive. The stable and reproducible behavior of micellar mobile phase allows the accurate prediction of the retention of solutes with a model that can also be used to optimize the separation of mixture of solutes (22).

MLC has proved to be a useful technique in the determination of diverse groups of compounds (23-30).

**Figure 1:**
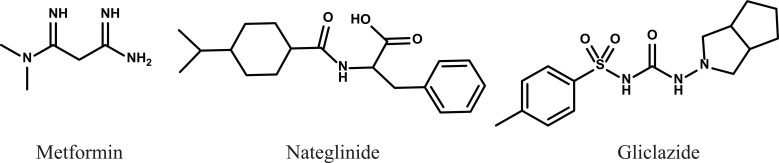
Structural formula of Metformin (MF), Nateglinide (NT) and Gliclazide (GL).

In the present work a sensitive method was developed and validated for the simultaneous determination of three antidiabetic drugs Metformin (MT), Nateglinide (NT) and gliclazide (GL) in their pharmaceutical preparations. Compounds under investigation possess different chemical structures are shown in Fig. 1.

## EXPERIMENTAL

### Materials and reagents

MT was purchased from Merck (Darmsrdat, Germany), NT was obtained from Toronto research chemicals, (Toronto, Canada), while GL pure sample was kindly provided by Pharco Pharmaceutical Co. (Alex, Egypt). Acetonitrile, methanol, n- propanol (all HPLC grade) and Triethylamine (TEA) were purchased from Riedel-de Haen (Seelze, Germany). Orthophosphoric acid for analysis was obtained from Prolabo (Paris, France). Tablets containing MT (Glucophage) & NT (Starlix) labeled to contain 120 mg NT, Starlix Combi labeled to contain MT 500 mg & NT120 mg, product of Novartis, tablets containing GL (Diamicron) labeled to contain 80 mg GL, product of Servier, were obtained from the local market. The micellar mobile phase, standard solution of antidiabetic drugs and pharmaceutical dosage forms were fitered through 0.4 μm nylon membranes (Micron Separations, Westboro, MA, USA).

### Instrumentation and chromatographic conditions

The chromatographic conditions were carried out in the isochratic mode using a mixture of SDS (0.12 mol.ml^-1^) and n-propranol (10%), triethylanine 0.3% adjusted to pH5.6 using orthophosphoric acid. The column Nucleosil C18, 4.6 × 150 mm, 5 μm, Macherey-Nagel GmbH & Co. KG, Duren-Germany) was equilibrated with the mobile phase for 30 min. at a flow rate of 1.0 mL.min^-1^. Each sample was injected by using 20 μL loop injector. The signal was measured by UV detector at 254 nm at ambient temperature.

### Standard solutions

Stock solution containing 100 μg.mL^-1^ of each of MT, NT, GL were prepared in methanol and stored at 4°C.The working solutions in the range (0.8-25 μg.mL^-1^) were freshly prepared by dilution with the mobile phase. The solutions were kept in the refrigerator and were found to be stable for one week.

**Construction of calibration curve.** Working solutions containing (0.8-25.0) μg.mL^-1^ of MT, NT and GL were prepared by serial dilution of the standard solutions with the mobile phase. Twenty μL aliquots were injected (triplicate) and eluted with the mobile phase under the previously described chromatographic conditions. The average peak areas of MT, NT and GL were plotted versus the corresponding concentration in μg.mL^-1^ to obtain the calibration graphs. Alternatively, the corresponding regression equations were derived. The results obtained were compared with those obtained by comparison methods (31-33).

**Analysis of pharmaceutical preparations.** Pharmaceutical preparations considered in this work were in the form of tablets, so for the analysis, ten tablets were weighed, grounded and homogenized to fine powder. An accurately weighed amount of the powder equivalent to 100 mg of the cited drugs, transferred to 100 mL volumetric flask dissolved in 50.0 mL methanol, sonicated for 10 min, diluted to volume with methanol and filtered, then a solution containing 20 μg.mL^-1^ was prepared from this solution by dilution with the mobile phase. The nominal content of the pharmaceutical preparation were calculated using the corresponding regression equations.

## RESULTS AND DISCUSSION

**Figure 2:**
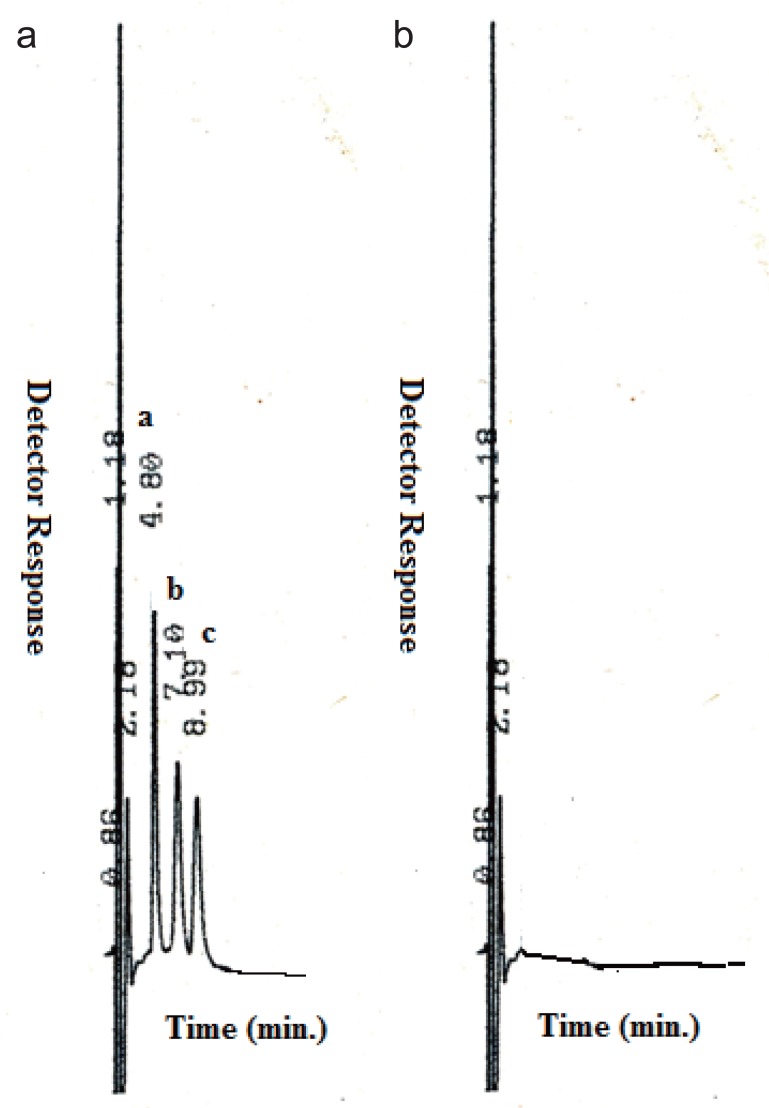
a: Typical chromatogram for the separation of Metformin (a) (10 μg/mL), Natiglinide (b) (20 μg/mL), Gliclazide (c) (20 μg/mL ) using micellar mobile phase. Chromatographic system: column, mobile phase, 0.12 M SDS, 10% n-propanol, 0.3 TEA, in 0.02 M phosphoric acid , pH5.6. Flow rate 1 mL/ min, UV detection at 254 nm: column temperature ambient; b: Chromatogram of placebo containing tablets exipients without active ingredients.

The optimum separation of the three antidiabetic drugs using a mobile phase consists of 0.12 M SDS, 10% n-propanol, and 0.3% TEA in 0.02 M phosphoric acid of pH5.6 was achieved in a short chromatographic run in time less than 10 min., MT (t_R_= 4.8 min), NT (t_R_=7.1) and GL (t_R_=8.99). Fig. 2a shows the obtained chromatogram of the three antidiabetic drugs.

### Optimization of the chromatographic conditions

The chromatographic method was optimized by changing the composition of the mobile phase.

**Figure 3:**
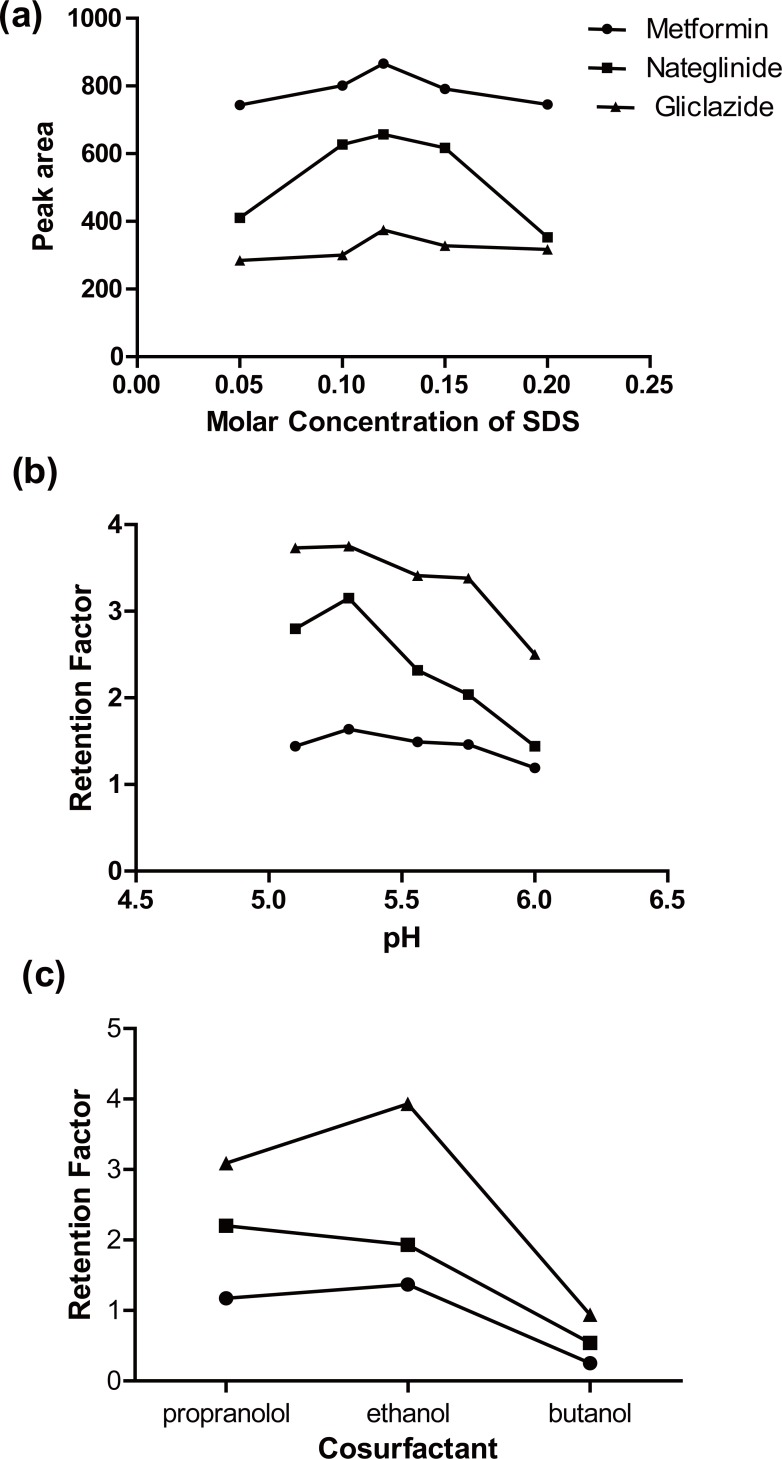
a) Peak area of metformin (●), natiglinide (▪) and gliclazide (▲) vs. different conc of SDS using micellar mobile phase consisting of different SDS molar concentration, 10% n-propanol, 0.3% triethylamine, in 0.02 M phosphoric acid, pH5.6; b) Retention factors of metformin (●), natiglinide (▪) and gliclazide (▲) vs. different pH using micellar mobile phase consisting of 0.12 M SDS, 10% n-propanol 0.3% triethylamine, in 0.02 M phosphoric acid of different pH values; c) Retention factors of metformin (•), natiglinide (▪) and gliclazide (▲) vs. different cosurfactants using micellar molile phase consisting of 0.12 M SDS, 10% different cosurfactants, 0.3% triethylamine in 0.02 M phosphoric acid pH5.6.

**The concentration of surfactants.** The effect of SDS concentration on retention time and detector response (as peak area) was investigated using micellar mobile phase containing SDS concentration from 0.05 to 0.2 M .It was found that increase the concentration of SDS decrease the retention factor of the three antidiabetic drugs continuously all over the investigated range due to their distribution into the surface of the droplets which run with the speed of the mobile phase. Meanwhile, increasing SDS concentration up to 0.12 M increased peak area of the three drugs further increase in SDS concentration up to 0.2 M decrease peak area of the three drugs Fig. 3a. A concentration of 0.12 M was found to be suitable for separation as it provides adequate elution time and selectivity.

**Effect of pH.** The pH of the mobile phase was changed in the interval from 5.1 to 6.0 using increasing amounts of TEA in phosphoric acid. The retention factors of the three drugs were plotted against different pH values as illustrated in Fig. 3b. It was observed that the three drugs are well separated with reasonable retention time at pH range (5.6-5.8). The three drugs differ in hydrophobicity and dissociation constants as expressed by their logP (octanol/water) and pka values respectively. Metformin has log *P* value of -2.6 and two pKa values of 2.8 & 11.5 (34). Nateglinide and gliclazide have pKa of 3.1 and 5.8 respectively. Thus ionization of the three drugs will decrease with increase pH.

**Effect of cosurfactant.** The effect of cosurfactant on the selectivity of the method was studied by replacing 10% n-propanol with either ethanol or 1-butanol. The retention factors of the three antidiabetic drugs are given in Fig. 3c as a function of the cosurfactants investigated and major difference in selectivity was observed. Ethanol and 1-butanol provided reasonable resolution of the three peaks. Upon using 10% propanolol separation is achieved with reasonable retention time.

**Effect of flow rate.** The effect of flow rate of the mobile phase on the separation and separation and resolution of the three antidiabetic drugs was investigated over the range from 0.8-1.2 mL/min. Flow rate of 1.0 mL/min was chosen for good separation at reasonable time.

## VALIDATION OF THE METHOD

The method was validated for Linearity, accuracy and precision, limit of detection (LOD), limit of quantification (LOQ), specificity, according to the ICH guideline Q1A (35).

### Linearity

A linear relationship was established by plotting peak area ratio for the analytes against their concentrations, the regression equations were determined over the concentration range of 0.4-16, 0.8-16 and 1-40 μg.mL^-1^ for MT, NT, GL respectively.

P = 5.8 × 10^-4^ + 0.233 C for NT (GL as internal standard)

P = 3.99 + 1.176 C for MT (GL as internal standard)

P = 0.052 + 0.52 C for GL ( MT as internal standard)

where P is peak area ratio, C is concentration of drug in μg.mL^-1^.

The high values of the correlation coefficient (r^2^-value>0.9999) with small intercept indicate good linearity of the calibration graphs. Statistical analysis of data gave small values of the standard deviation of the residuals (S_y/x_), slope (S_b_) and of intercept (S_a_), and the % relative error (Table 1), indicating low scattering of the points around the calibration graphs.

**Table 1:** Analytical data for the simultaneous HPLC determination of Metformin, nateglinide and gliclazide using micellar mobile phase

Parametrs	Metformin	Nateglinide	Gliclazide

Conc.range (μg/mL)	0.4-16	0.8-16	1-40
Correlation coefficient (r)	0.9999	0.9999	0.9998
Slope	1.176	0.233	0.052
Intercept	3.99	5.89 × 10^-4^	0.52
LOQ (μg/mL)	0.143	3.49 × 10^-3^	0.109
LOD (μg/mL)	0.047	1.15 × 10^-3^	0.036
S_y/x_	4.88	4.71 × 10^-3^	0.132
S_a_	0.0169	8.13 × 10^-5^	0.0057
S_b_	0.35	3.44 × 10^-4^	0.0037
%RSD	0.82	0.259	0.92
%Er	0.34	0.105	0.35

LOQ, Limit of quantification; LOD, Limit of detection; S_y/x_, Standard deviation of the residuals; S_a_, Standard deviation of the intercept; S_b_, standard deviation of the slope; %RSD, Percentage relative standard deviation for ten replicate samples; % Er, Percentage relative error for ten replicate samples.

### Accuracy and precision

To test the accuracy of the proposed MLC method, it was applied to the determination of pure samples of the antidiabetic drugs over the concentration range cited in Table 1. The results obtained were in good agreement with those obtained using comparison methods (31-33). Statistical evaluation of the results using the student’s t test and the variance ratio *F*-test revealed no significant difference between the performance of the two methods regarding accuracy and precision (Table 2). The intra-day precision of the method was determined by analyzing triplicate concentrations of each concentration level of the analytes in their pharmaceutical preparations on one day. The inter-day precision was evaluated over three separate days by analyzing different samples of analytes at each level. The obtained results for intra-day and inter-day are summarized in Tables 3 & 4 respectively. The repeatability and reproducibility in the proposed method were fairly good, as indicated by the small values relative standard deviation (% RSD), and error (% Er) Table 1.

**Table 2:** Application of proposed HPLC method using micellar mobile phase to the determination of metformin, nateglinide and gliclazide in pure form

	Taken (μg/mL)	Found (μg/mL)	% of declared amount	Comparison methods (31-33)

Metformin	0.4	0.397	99.25	100.58
	0.8	0.800	100.0	99.25
	2.0	2.010	100.5	100.5
	4.0	3.997	99.93	
	10.0	9.968	99.68	
	16.0	16.00	100.0	
X′			99.89	100.11
± SD			± 0.82	± 0.74
*t*			0.63 (2.447)	
F			1.23 (19.33)	
Nateglinide	0.8	0.804	100.5	101.7
	1.0	1.009	100.9	101.2
	2.0	2.00	100.0	100.98
	4.0	3.998	99.95	
	10.0	9.99	99.99	
	16.0	16.01	100.06	
X′			100.23	101.29
± SD			± 0.38	± 0.37
*t*			0.76 (2.447)	
F			1.05 (19.33)	
Gliclazide	1.00	0.990	99.99	101.3
	2.00	2.031	101.55	100.9
	10.0	10.710	101.70	100.5
	16.0	15.980	99.88	
	20.0	19.944	99.720	
	30.0	30.360	101.20	
	40.0	39.889	99.72	
X′			100.53	100.9
± SD			± 0.90	± 0.4
*t*			0.65 (2.306)	
F			5.06 (9.55)	

Each result is the average of three separate determinations; Values between parentheses are the tabulated *t* and F values at *P*=0.05, respectively.

**Table 3:** Intra-day accuracy and precision for determination of Metformin, nateglinide and gliclazide in dosage forms using micellar mobile phase

Pharmaceutical preparation	% recovery repeatability 1 μg/mL	% recovery repeatability 2 μg/mL	% recovery repeatability 10 μg/mL

1) Starlix tablets Combi (500 mg metformin & 120 mg nateglinide)			
a) Metformin	99.50	99.40	99.40
	100.40	99.50	100.10
	99.76	98.90	100.20
Mean found (%)	99.89	99.30	99.90
± S.D.	0.46	0.346	0.435
RSD%	0.46	0.348	0.436
b) Nateglinide	99.45	100.2	99.5
	99.00	99.5	100.6
	100.60	99.8	100.4
Mean found (%)	99.68	99.8	100.16
± S.D.	0.83	0.35	0.59
RSD%	0.83	0.35	0.59
2) Diamicron tablets (Gliclazide 500 mg/tablet)			
	99.89	98.5	100.9
	100.70	100.2	99.2
	100.90	99.1	99.6
Mean found (%)	100.49	99.2	99.9
± S.D.	0.53	0.86	0.89
RSD%	0.53	0.87	0.89

Each result is the average of three separate determinations.

**Table 4:** Inter-day accuracy and precision for determination of Metformin, nateglinide and gliclazide in dosage forms using a miceller mobile phase

Pharmaceutical preparations	% recovery repeatability 1 μg/mL	% recovery repeatability 2 μg/mL	% recovery repeatability 10 μg/mL

1) Starlix tablets Combi (500 mg metformin & 120 mg nateglinide)			
a) Metformin	99.8	100.8	100.9
	98.9	101.2	100.1
	99.0	100.5	99.8
Mean found (%)	99.2	100.8	100.3
± S.D.	0.49	0.35	0.57
RSD%	0.49	0.34	0.56
b) Nateglinide	98.7	100.7	99.4
	99.5	99.5	100.3
	100.9	100.4	100.8
Mean found (%)	99.6	100.2	100.16
± S.D.	0.95	0.62	0.71
RSD%	0.95	0.61	0.70
2) Diamicron tablets (500 mg gliclazide/tablet)			
	100.9	101.2	100.4
	100.3	99.3	100.9
	99.0	99.8	101.2
Mean found (%)	100.07	100.1	100.8
± S.D.	0.97	0.88	0.40
RSD%	0.97	0.87	0.39

Each result is the average of three separate determinations.

### Limits of quantification (LOQ) & limit of detection (LOD)

(LOQ) & (LOD) were determined according to ICH guideline Q2 (R1) (35). LOQ and LOD were calculated according to the following equations.

LOQ = 10 σ/S

LOD = 3.3 σ/S

where σ is the standard deviation of y-intercepts of the regression lines (standard deviation of the response) and S is the slope of the calibration curve. The results are summarized in (Table 1).

### Specificity

The method specificity was proved as no interference was observed from tablet excipients. A placebo containing common tablets excipients omitting the active ingredients was prepared and 20 μL was injected under the described chromatographic conditions for the assay. As revealed from Fig. 2b, these matrix components did not show any interfering peaks at the retention times of the antidiabetic drugs.

## ASSAY OF DOSAGE FORMS

**Figure 4:**
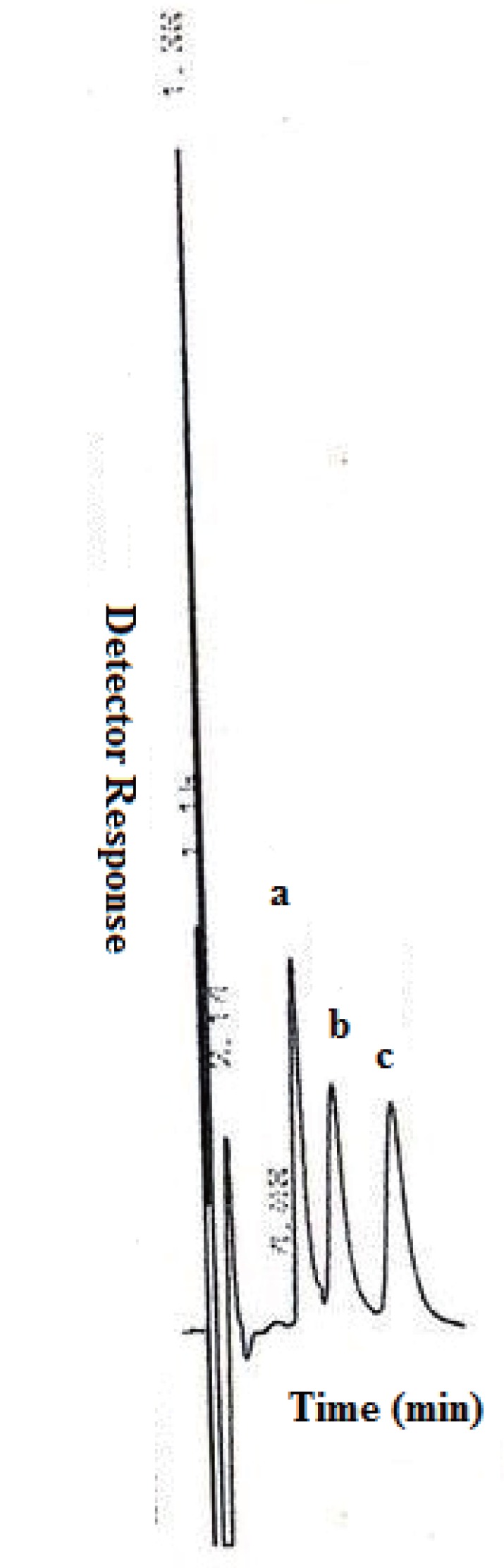
Representative chromatogram showing a) 10 μg/mL Glucophage tablet; b) 10 μg/mL Starlix tablet; c) 10 μg/mL Diamicron tablet under described chromatographic conditions.

The proposed method was successfully applied to the assay of MT & NT & GL in commercial tablets Fig. 4. The average percent recoveries of different concentrations were based on the average of three replicate determinations. Recovery data obtained from the developed MLC method were statistically compared with those of the comparison methods (31-33) using the Student’s t-test and the variance ratio F-test. The results shown in Table 5 were in good agreement with those obtained with comparison methods.

**Table 5:** Application of the proposed HPLC method using micellar mobile phase to the determination of metformin, nateglinide and gliclazide in dosage forms

Preparation	Taken (μg/mL)	Found (μg/mL)	% of declared amount	Comparison methods (31-33)

Glucophage tablets (Metformin120 mg/tab)				
	2.0	1.98	99.0	99.8
	4.0	3.97	99.25	100.5
	10.0	10.06	100.6	99.9
	16.0	15.97	99.8	
X′			99.66	100.07
± SD			± 0.71	± 0.38
t			2.3 (2.57)	
F			0.29 (9.55)	
Starlix tablets (Nateglinide 500 mg/tab)				
	2.0	2.01	100.5	100.35
	4.0	3.99	99.75	100.45
	10.0	10.07	100.7	99.6
	16.0	15.9	99.38	
X′			100.08	100.13
± SD			± 0.62	± 0.46
t			1.04 (2.57)	
F			0.74 (9.55)	
Diamicron tablets (500 mg gliclazide/tab)				
	10.0	10.1	101.0	99.75
	16.0	16.06	100.38	99.85
	20.0	19.95	99.75	100.5
	30.0	29.97	99.90	
X′			100.31	100.03
± SD			± 0.5	± 0.41
t			0.057 (2.57)	
F			0.76 (9.55)	

Each result is the average of three separate determinations; Values between parentheses are the tabulated *t* and F values at *P*=0.05, respectively.

## SOLUTION STABILITY

The stability of the stock solution was determined by quantification of antidiabetic drugs and comparison to freshly prepared standard solutions. No significant change was observed in standard solution response, relative to freshly prepared standard. Similarly, the stability of the mobile phase was checked. The results obtained in both cases proved that the sample solution and mobile phase used during the assay were stable up to 7 days.

## CONCLUSION

The proposed micellar liquid chromatography method for the determination of metformin, nateglinide and gliclazide was found to be simple, sensitive and precise. The method can be used for determining the three drugs in their pharmaceutical preparations in single short time chromatographic run.
